# Liposomal Nanoparticles of a Spleen Tyrosine Kinase P-Site Inhibitor Amplify the Potency of Low Dose Total Body Irradiation Against Aggressive B-Precursor Leukemia and Yield Superior Survival Outcomes in Mice^☆^

**DOI:** 10.1016/j.ebiom.2015.04.005

**Published:** 2015-04-11

**Authors:** Fatih M. Uckun, Dorothea E. Myers, Jianjun Cheng, Sanjive Qazi

**Affiliations:** a Children's Center for Cancer and Blood Diseases, Children's Hospital Los Angeles (CHLA), Los Angeles, CA 90027, USA; b Department of Pediatrics, University of Southern California Keck School of Medicine (USC KSOM), Los Angeles, CA 90027, USA; c Norris Comprehensive Cancer Center, University of Southern California Keck School of Medicine (USC KSOM), Los Angeles, CA 90027, USA; d Department of Materials Science and Engineering, University of Illinois at Urbana–Champaign (UIUC), Urbana, IL 61801, USA; e Bioinformatics Program, Gustavus Adolphus College, 800 W College Avenue, St. Peter, MN 56082, USA

**Keywords:** BPL, B-precursor acute lymphoblastic leukemia, LNP, liposomal nanoparticles, SYK, spleen tyrosine kinase, Leukemia, Bone marrow transplantation, Total body irradiation, Radiation resistance, Personalized medicine, Precision medicine, Cancer

## Abstract

This study was designed to improve the efficacy of radiation therapy against radiation-resistant leukemia. We report that the potency of low dose radiation therapy against B-precursor acute lymphoblastic leukemia (BPL) can be markedly enhanced by combining radiation with a liposomal nanoparticle (LNP) formulation of the SYK-P-site inhibitor C61 (“C61-LNP”). C61-LNP plus low dose total body irradiation (TBI) was substantially more effective than TBI alone or C61-LNP alone in improving the event-free survival outcome NOD/SCID mice challenged with an otherwise invariably fatal dose of human ALL xenograft cells derived from relapsed BPL patients. C61-LNP plus low dose TBI also yielded progression-free survival, tumor-free survival and overall survival outcomes in CD22ΔE12 × BCR–ABL double transgenic mice with advanced stage, radiation-resistant BPL with lymphomatous features that were significantly superior to those of mice treated with TBI alone or C61-LNP alone.

## Highlights

• Liposomal nanoparticles of C61 kill radiation-resistant leukemia cells
• Liposomal nanoparticles of C61 potentiate TBI against human leukemia cells
• Liposomal nanoparticles of C61 plus TBI improve the survival outcome of leukemic mice.

## 1 Introduction

Spleen tyrosine kinase (SYK) is a cytoplasmic protein tyrosine kinase with multiple important regulatory functions in B-lineage lymphoid cells (Mocsai et al., 2010, Uckun et al., 2010a, Uckun et al., 2012, Uckun et al., 2014, Uckun and Qazi, 2010). SYK functions as a master regulator of apoptosis controlling the activation of the phosphatidylinositol-4,5-bisphosphate 3-kinase (PI3-K), nuclear factor kappa B (NFκB), and signal transducer and activator of transcription 3 (STAT3) pathways that have been implicated in radiation resistance of BPL cells (Uckun and Qazi, 2010). We recently identified the pentapeptide mimic 1,4-bis (9-*O*-dihydroquinidinyl) phthalazine/hydroquinidine 1,4-phathalazinediyl diether (“compound 61”) (C61) as a highly selective and potent inhibitor targeting the substrate binding P-site of SYK (Uckun et al., 2010a, Uckun et al., 2010b). We developed a multifunctional liposomal nanoparticle (LNP) formulation of C61 as a nanoscale pharmaceutical modality against B-precursor acute lymphoblastic leukemia (BPL) (Uckun et al., 2013). Our study provides preclinical proof of principle that the in vivo anti-leukemic potency of low dose total body irradiation (TBI) regimens can be significantly augmented by C61-LNP. Further development of C61-LNP as a selective TBI potentiator may provide the foundation of more effective TBI-based conditioning regimens for BPL patients undergoing hematopoietic stem cell transplantation (HSCT).

## 2 Methods

### 2.1 Analysis of Gene Expression

We compiled 6 archived gene expression profiling datasets that measured expression from B- and T precursor ALL patients hybridized to the Human Genome U133 Plus 2.0 Array (GSE11877, N = 207; GSE13159 N = 823; GSE13351 N = 107; GSE18497, N = 82, GSE28460, N = 98; GSE7440, N = 99; Total, N = 1416). To enable comparison of samples across studies, a normalization procedure was performed that merged the raw data from the 6 datasets (CEL files). Perfect Match (PM) signal values for probesets were extracted utilizing raw CEL files matched with probe identifiers obtained from the Affymetrix provided CDF file (HG-U133_Plus_2.cdf) implemented by Aroma Affymetrix statistical packages run in R-studio environment (version 0.97.551, R-studio Inc., running with R 3.01). The PM signals were quantified using Robust Multiarray Analysis in a 3-step process, including RMA background correction, quantile normalization, and summarization by Median Polish of probes in a probeset across 1416 samples (RMA method adapted in Aroma Affymetrix). RMA background correction estimates the background by a mixture model, whereby the background signals are assumed to be normally distributed and the true signals are exponentially distributed. Normalization across all 6 studies and 1416 samples was achieved using a two-pass procedure. First the empirical target distribution was estimated by averaging the (ordered) signals over all arrays, followed by normalization of each array toward this target distribution. After removing normal cells from analysis, we focused our analysis of gene expression datasets on leukemic cells (N = 1342) to include BPL (N = 207 from GSE11877, N = 575 from GSE13159, N = 92 from GSE13351, N = 54 from GSE18497, N = 98 from GSE28460 and N = 99 from GSE7440) and T-precursor ALL (N = 174 from GSE13159, N = 15 from GSE13351, N = 28 from GSE18497). Pearson pairwise correlations were performed for 38 probesets including SYK, STAT3, SYK-dependent STAT3 target genes (KLF4, SPRY2, CYR61, BIRC5 and BCL2L1) (Uckun et al., 2010a), and SYK-dependent anti-apoptotic genes (DAD1, GCLC, HSPA5, TCF7L2, TNFAIP8) (Uckun and Qazi, 2010) using this RMA normalized database. Correlation coefficients (r) were determined between all probeset pairs and hierarchical cluster analysis was applied to the matrix of correlation coefficient for both rows and columns of probeset identifications using the average distance metric to visualize sub-clusters of expression profiles (JMP Software, SAS, Cary, NC). We also examined archived gene expression profiling (GEP) data (GSE18497 and GSE28460) on initial diagnostic bone marrow samples from 48 BPL patients, who experienced an early relapse (< 36 months after diagnosis) vs. 28 BPL patients, who experienced a late relapse (≥ 36 months after diagnosis) to determine if their expression levels of the SYK–STAT3 pathway genes differed (2-sample T-test, unequal variance correction). A one-way hierarchical clustering technique was utilized to visualize similar expression of significantly affected probesets for newly diagnosed samples comparing “Early” versus “Late” relapse patients (calculated using the average distance metric). Dendrograms were drawn to illustrate similar gene-expression profiles from joining pairs of closely related gene expression profiles, whereby genes and samples joined by short branch lengths showed the most similarity in expression profile across patient samples and genes (JMP Software, SAS, Cary, NC). The heat map represents the color-coded expression value reported as mean centered expression level relative to log_2_ transformed RMA expression levels mean centered to late relapse samples.

### 2.2 LNP of 1,4-Bis (9-*O*-Dihydroquinidinyl) Phthalazine/Hydroquinidine 1,4-Phathalazinediyl Diether (C61-LNP)

The C61-LNP formulation was prepared using the standard thin film evaporation method (Uckun et al., 2013). C61 was entrapped within the LNP using a pH gradient with the help of lactobionic acid to establish a low pH inside the LNP. The C61-LNP had a diameter of 136.3 ± 1.2 nm (mean ± SEM); a negative surface charge with a Zeta potential of − 12.1 ± 0.8 mV (mean ± SEM) and contained 8.7 ± 0.1 mg/mL (mean ± SEM) C61 (Uckun et al., 2013; Supplementary Fig. 1). It inhibited the constitutive activity of SYK (but not Bruton's tyrosine kinase) in the BCR–ABL^+^ BPL cell line ALL-1 in a concentration-dependent fashion and prevented the CD19-mediated activation of SYK without affecting SYK protein expression levels (Uckun et al., 2013).

### 2.3 Xenograft Samples

The xenografts were established using primary cells from relapsed pediatric BPL patients (Uckun et al., 2010a, Uckun et al., 2013, Uckun and Qazi, 2010). The secondary use of leukemic cells for subsequent molecular studies did not meet the definition of human subject research per 45 CFR 46.102 (d and f) since it did not include identifiable private information, and it was approved by the IRB (CCI) at the Children's Hospital Los Angeles (CHLA).

### 2.4 Irradiation of Cells and Mice

NOD/SCID mice and CD22ΔE12 × BCR–ABL double-Tg mice with advanced leukemia were placed in autoclaved 2 L Pyrex Griffin glass beakers (VWR, Radnor, PA) and irradiated with single dose TBI (2 Gy for NOD/SCID mice/4 Gy for double-Tg C57BL/6 mice) delivered at 106 cGy/min using a self-shielded Cs-137 irradiator (Mark I Irradiator-68A, JL Sheperd & Associates, San Fernando, CA), as previously reported (Uckun et al., 2015). Cells were irradiated with 2 Gy γ-rays in a single exposure using the Mark I Cs-137 irradiator (Uckun et al., 2013, Uckun et al., 2015a, Uckun et al., 2015).

### 2.5 SCID Mouse Xenograft Model of Human BPL

We used a NOD/SCID mouse model of relapsed human BPL (Uckun et al., 2013, Uckun et al., 2015a). NS mice (NOD.CB17-*Prkdc^scid^* J; 4–6 weeks of age at the time of purchase, female) were obtained from the Jackson Laboratory (Sacramento, CA). The research was conducted according to the Institutional Animal Care and Use Committee (IACUC) Protocol # 280-12 that was approved on 7-10-2012. The specific pathogen-free (SPF) environment for immunodeficient NS mice was ensured by the use of Micro-Isolator cages, which were autoclaved, complete with rodent chow and hardwood Sani-Chips for bedding. Water was provided ad libitum and was also autoclaved as well as supplemented with Bactrim or Septra (0.89 mg per mL sulfamethoxazole, 0.18 mg per mL trimethoprim) by adding 22.75 mL of Bactrim or Septra to each liter of water once per week as prophylaxis. A light and dark cycle of 12 h each was strictly adhered to, as was a room temperature of 70–75 °F. Animals remained within the confines of the Micro-Isolators except for scheduled cage changes and treatments, which were performed in a laminar flow hood. Ibuprofen was used as a pain reliever to reduce the discomfort associated with treatment or inoculation of leukemia cells. In some experiments, NOD/SCID mice (6–8 weeks old, female, same age in all cohorts in each independent experiment) were inoculated intravenously with BPL xenograft cells (1 × 10^6^ leukemia cells in Xeno Case #12; 2 × 10^6^ leukemia cells in Xeno Case #14) in 0.2 mL PBS via tail vein injection with a 27-gauge needle. All NOD/SCID mice were genetically identical, of the same age, and in each experiment all mice were inoculated with the same number of BPL cells from the identical BPL xenograft clone. This statistical equivalency of mice allowed the use of a pseudo-randomization convenience allocation to assign mice to identified cages. For random treatment allocation, cages were randomly selected to receive one of the specified treatments. We applied concealment of treatment allocation and blind outcome assessment to reduce the risk of bias in our conclusions. Daily health care assessments were performed by animal care technicians not involved in the treatment assignments or treatments who also made the determinations about which of the mice needed to be electively sacrificed to meet the humane endpoints criteria in laboratory animal experimentation. Investigators did not participate in individual health status or outcome assessments. Mice were treated with C61-LNP, low dose TBI, or C61-LNP + low dose TBI according to the following schedules: (i) 3-day schedule = Days 1–3: C61-LNP, 80 mg/kg/day i.v. 2 Gy single dose TBI on Day 2, 1 h post C61-LNP injection in the C61-LNP + TBI group and (ii) 5-day schedule = Days 1–5: C61-LNP, 80 mg/kg/day i.v. 2 Gy single dose TBI was administered 1 h post C61-LNP injection on Day 5. Controls included untreated mice and mice treated with D5W. Mice were monitored daily and electively euthanized at the indicated time points by CO_2_ asphyxia. At the time of their death or elective sacrifice, mice were necropsied to confirm leukemia-associated splenomegaly. Spleens of mice were removed, measured, and cell suspensions were prepared for determination of mononuclear cell counts and immunophenotyping. Multiple organs were preserved in 10% neutral phosphate buffered formalin, and processed for histologic sectioning. For histopathologic studies, formalin fixed tissues were dehydrated and embedded in paraffin by routine methods. Glass slides with affixed 4–5 micron tissue sections were prepared and stained with Hematoxylin and Eosin (H&E). The brain, liver, kidney and bone marrow were examined for their leukemic involvement. Images were taken with an EVOS XL Core Light Microscope (AMG, Bothell, WA) using 20 × and 40 × objectives or a Nikon Eclipse Ci camera (Melville, NY) equipped with Nikon's Digital Sight DS-U3 microscope camera controller and Nikon's advanced imaging software suite NIS-Elements. For the analysis of the NOD/SCID mouse xenograft data on the in vivo potency of various treatments, event-free survival (EFS) times were measured from the day of inoculation of xenograft cells to the day of death or killing. The probability of survival was determined and the event-free interval curves were generated using the Kaplan–Meier product limit method, as in previous studies (Uckun et al., 2013, Uckun et al., 2015a, Uckun et al., 2015b). Log-rank tests were performed to compare differences in median survival estimates between all groups and pairwise comparison of the individual treatment groups. In experiments aimed at evaluating the effects of C61-LNP + radiation (RAD) on the leukemia initiating cells (LICs) (i.e. putative leukemic stem cell fractions capable of engrafting and causing overt leukemia in NOD/SCID mice) in the BPL xenograft samples, leukemia cells (cell density: 2 × 10^6^ cells/mL) isolated from spleens of xenografted mice challenged with primary leukemic cells from 2 pediatric BPL patients in relapse were (i) irradiated with 2 Gy γ-rays (N = 11), (ii) treated for 24 h at 37 °C with C61-LNP at a concentration of 30 μg/mL (based on C61 content), (iii) treated with 2 Gy γ-rays + 30 μg/mL C61-LNP (24 h incubation at 37 °C) (N = 4), or (iv) left untreated for 24 h at 37 °C and then reinjected into NOD/SCID mice. Mice injected with in vitro treated xenograft cells were monitored daily in the same fashion as NOD/SCID mice that were subjected to systemic treatments. Mice were electively euthanized by CO_2_ asphyxia when any mouse developed morbidity.

### 2.6 Transgenic (Tg) Mice With Murine B-Precursor Leukemia

The CD22ΔE12-transgenic (Tg) mouse model of BPL was recently described (Uckun et al., 2015b). CD22ΔE12 × BCR–ABL double-Tg mice were established by breeding commercially obtained male BCR–ABL (p190) Tg founder mice (B6.Cg-Tg (BCR/ABL) 623Hkp/J, Jackson Labs) with female CD22ΔE12-Tg mice (Uckun et al., 2015).

### 2.7 Evaluation of the Efficacy of the Systemic C61-LNP + Low Dose TBI Regimen in CD22ΔE12 × BCR–ABL Double Transgenic Mice With Advanced B-Precursor Leukemia

CD22ΔE12 × BCR–ABL double-Tg mice spontaneously develop fatal B-precursor leukemia with lymphomatous features at a median of 78 days. A prospective power analysis (one-sample proportions using the exact method based on a binomial distribution, one-sided test; JMP, SAS, Cary, NC) showed that a sample size of 5 mice per treatment group would be required to demonstrate an increase in the proportion of CD22ΔE12 × BCR–ABL double-Tg mice surviving at 4 weeks from the onset of symptomatic leukemia from a baseline of 0% to 45% with a specific treatment at a 5% significance level and 95% power. Therefore, it was determined that using a group size of 5 mice would make a comparative study sufficiently powered to detect treatment-related large effect sizes. Mice developing BPL were randomly assigned to one of the 4 treatment groups (viz.: No treatment, 4 Gy TBI, C61-LNP (80 mg/kg) alone, and TBI + C61-LNP), as previously described (Uckun et al., 2015). Treatment commenced within 2 days of mice becoming symptomatic and/or developing measurable masses of leukemic cells. C61-LNP was administered i.v. either as a single 80 mg/kg/day dose or as a multiple dose regimen daily for 3 or 9 consecutive days (80 mg/kg/day). TBI (4 Gy) was administered as a single dose 1 h after injection of C61-LNP on Day 3 in the multiple dose regimen or on Day 1 in the single dose regimen. The masses were photographed using an iPhone 4S (Los Angeles, CA) equipped with an 8-megapixel iSight camera and the dimensions were measured at indicated time points in order to determine the effect of the treatments on disease progression. Since the same mice were not measured on the same days, we observed day to variation across days due to sampling differences and not due to variation in tumor volumes for any given mouse. This statistical artifact was taken into account using a Mixed Model ANOVA to partition the variance due to treatment and time the measurements were taken (fixed factors), and multiple measurements taken from each individual mouse at different times (random factor). Randomization, concealment of treatment allocation and blind outcome assessment were used to reduce the risk of bias in our conclusions. Daily health care assessments were performed by animal care technicians not involved in the treatment assignments or treatments. Investigators did not participate in individual health status or outcome assessments. In addition, any unintended intergroup differences in initial tumor size were formally excluded by statistical comparisons, as previously reported (Uckun et al., 2015). Statistical analyses were performed by a bioinformatics expert (S.Q.) who was not involved in treatment assignments, treatments, or outcome assessments. The tumor-free survival (TFS) (duration of tumor-free interval), progression-free survival (PFS) (time from initiation of therapy to 10% increase in the longest diameter of tumor mass), and overall survival (OS) (time from onset of symptomatic leukemia to the day of death or killing) were determined for each treatment group. Significance of pairwise differences in median values between treatment groups for TFS and PFS times were assessed using non-parametric Wilcoxon tests (JMP software v10.02, SAS, Cary, NC). The probability of OS was determined and the event-free interval curves were generated using the Kaplan–Meier product limit method, as in previous studies (Uckun et al., 2013, Uckun et al., 2015a, Uckun et al., 2015b). Log-rank tests were performed to compare differences in median survival estimates between all groups and pairwise comparison of the individual treatment groups. Post-treatment tumor size (longest diameter) was normalized to Day 1 measurements. Tumor growth profiles of control mice and mice treated with the C61-LNP plus TBI regimen were compared using a repeated measures analysis of covariance controlling for heterogeneity between mice (REML method to partition experimental and mice variance components; JMP software v10.02, SAS, Cary, NC). The model was comprised of a fixed factor (“treatment”), time co-variate (“day”), interaction term (“treatment × day”) and a random factor (Mouse ID). F-test comparing the least square means for control versus C61-LNP plus TBI means calculated in the fixed factor was utilized to assess the significance of overall remission or reduction of tumor size (P-values < 0.05 deemed significant). The probability of survival (OS) was determined and the event-free interval curves were generated using the Kaplan–Meier product limit method, as in previous studies (Uckun et al., 2013, Uckun et al., 2015a, Uckun et al., 2015b, Uckun et al., 2015).

### 2.8 Statistical Analysis

Standard methods were used for statistical analysis of data (Uckun et al., 2013, Uckun et al., 2015a, Uckun et al., 2015). For the evaluation of the in vivo anti-leukemic activity of C61-LNP, the probability of survival was determined and the event-free interval curves were generated for each treatment group using the Kaplan–Meier product limit method, as previously reported (Uckun et al., 2013, Uckun et al., 2015). Log-rank tests were performed to compare differences in median survival estimates between all groups and pairwise comparison of pooled controls vs. test mice treated with C61-LNP, TBI, or C61-LNP + TBI. For the analysis of the in vitro potency of various treatments against LIC in xenograft specimens, we compared the mean spleen size and spleen cellularity of mice inoculated with xenograft cells that were subjected to the respective treatments prior to injection. Planned Linear Contrasts that were constructed from one-way ANOVA for spleen size and nucleated spleen cell counts (log_10_ transformed). P-values less than 0.05 were deemed significant if the False Discovery Rate was less than 10%. In CD22ΔE12 × BCR–ABL double-Tg mice, the post-treatment tumor size (longest diameter) was normalized to Day 1 measurements. Tumor growth profiles of control mice and mice treated with the C61-LNP + low dose TBI regimen were compared using a repeated measures analysis of covariance controlling for heterogeneity between mice (REML method to partition experimental and mice variance components; JMP Software v10.02, SAS, Cary, NC). The intergroup differences in TFS, PFS and OS were evaluated for statistical significance as previously reported (Uckun et al., 2015). Significance of pairwise differences in medians between treatment groups for TFS and PFS times was assessed using non-parametric Wilcoxon tests (JMP Software v10.02, SAS, Cary, NC). The probability of OS was determined and the event-free interval curves were generated using the Kaplan–Meier product limit method, as in previous studies (Uckun et al., 2013, Uckun et al., 2015a, Uckun et al., 2015). Log-rank tests were performed to compare differences in median survival estimates between all groups and pairwise comparison of the individual treatment groups.

### 2.9 Study Approval

The animal research in mice was conducted according to Institutional Animal Care and Use Committee (IACUC) Protocols 280-12 and 293-10 that were approved by the IACUC of CHLA. All animal care procedures conformed to the Guide for the Care and Use of Laboratory Animals (National Research Council, National Academy Press, Washington DC 1996, USA). BPL xenograft clones derived from deidentified patient specimens were used in the described experiments. The secondary use of leukemia cells for subsequent laboratory studies did not meet the definition of human subject research per 45 CFR 46.102 (d and f) since it did not include identifiable private information, and the corresponding research protocol CCI-10-00141 was approved by the CHLA IRB (CCI) (Human Subject Assurance Number: FWA0001914).

## 3 Results

### 3.1 Upregulated Expression of SYK-STAT3 Pathway Genes as a Predictor of Early Relapse

**Fig. 1:**
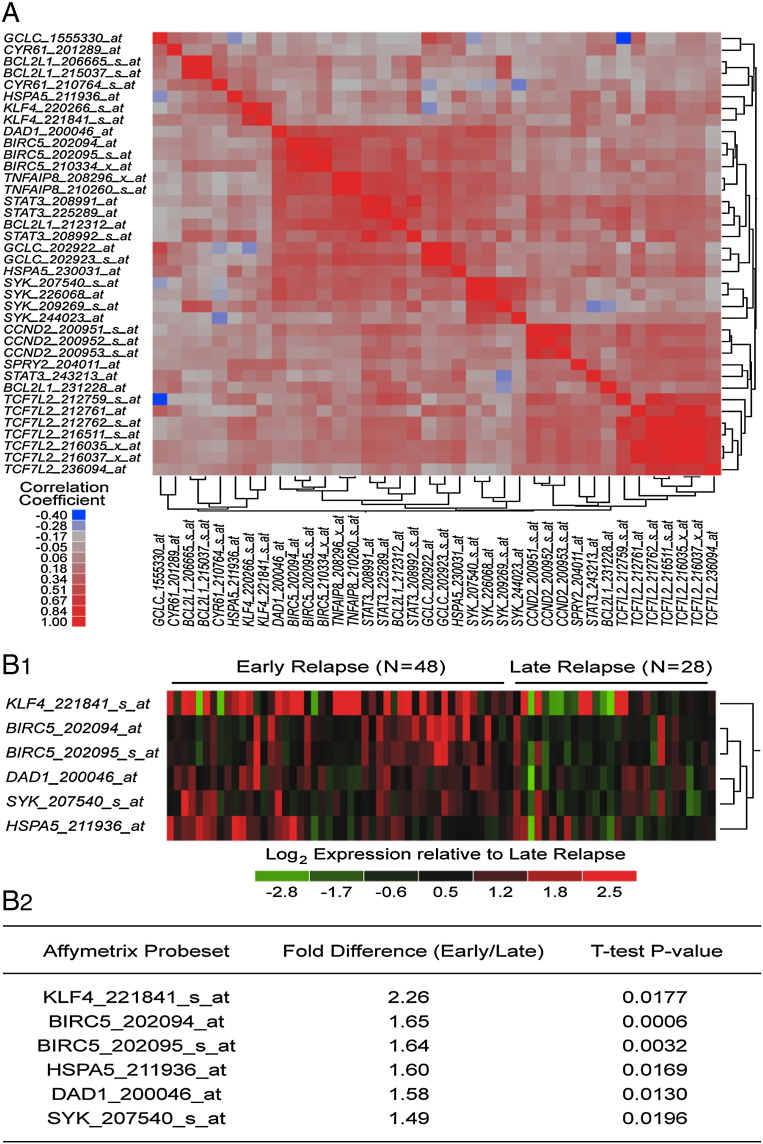
[A] Correlation between expression levels of SYK pathway genes and STAT3 target genes. SYK has been shown to regulate the activation of STAT3 and thereby the expression levels of STAT3 target genes. Publicly available gene expression datasets from 6 studies examining newly diagnosed B-cell precursor/B-lineage ALL (N = 207 from GSE11877, N = 575 from GSE13159, N = 92 from GSE13351, N = 54 from GSE18497, N = 98 from GSE28460 and N = 99 from GSE7440) and T-lineage ALL (N = 174 from GSE13159, N = 15 from GSE13351, N = 28 from GSE18497) were merged and normalized using the RMA method. Pearson pairwise correlations were performed for 38 probesets including SYK, STAT3 and SYK-dependent STAT3 target genes (*KLF4*, *SPRY2*, *CYR61*, *BIRC5* and *BCL2L1*) and SYK-dependent anti-apoptotic genes (*DAD1*, *GCLC*, *HSPA5*, *TCF7L2*, *TNFAIP8*). Correlation coefficients (r) were determined between all probeset pairs and hierarchical cluster analysis was applied to the matrix of correlation coefficient for both rows using the average distance metric to visualize sub-clusters of expression profiles that exhibited positive correlations and negative correlations between these probesets. Highly significant (P < 0.00001) pairwise correlations were observed for 431 out of the 703 possible pairs for 38 probesets examined. The majority of these highly significant correlations were probesets that exhibited positive correlations versus negative correlations between these probesets (368 positive correlations, 63 negative correlations). Pearson correlation coefficients were organized using a clustering algorithm to reveal highly positively co-regulated sets of probesets revealing a sub-cluster of 4 probesets for SYK, 3 probesets for STAT3, 2 probesets for TNFAIP8, 2 probesets for GCLC, 1 probeset for BCL2L1, 1 probeset for HSPA5, 3 probesets for BIRC5 and 1 probeset for DAD1 that were highly correlated across 1342 leukemia samples. [B] Primary leukemic blast cells in diagnostic bone marrow samples from BPL patients who experience an early relapse are characterized by upregulated expression of SYK-STAT3 pathway genes. [B1] A one-way hierarchical clustering technique was utilized to visualize similar expression of 6 significantly affected probesets for newly diagnosed samples comparing “Early” versus “Late” relapse patients. The heat map represents the color-coded expression value reported as mean centered expression level relative to log_2_ transformed RMA expression levels mean centered to late relapse samples (green to red depicting increasing levels of expression in early relapsed patients). [B2] Gene expression values for primary leukemic cells in diagnostic specimens from BPL patients who experienced an early (N = 48; time to relapse < 36 months) versus late relapse (N = 28; time to relapse ≥ 36 months) were compared using Student's T-test (unequal variance correction).

SYK has recently been shown to regulate the activation of the anti-apoptotic transcription factor STAT3 and thereby the expression levels of STAT3 target genes (Uckun et al., 2010a, Uckun and Qazi, 2010). We examined archived GEP datasets from 1342 primary ALL samples to determine if the expression levels of SYK pathway genes are correlated with the expression levels of the STAT3 target genes (Fig. 1A). Highly significant (P < 0.00001) pairwise correlations were observed for 431 out of the 703 possible pairs for the 38 probesets examined in this study. At least one of the SYK probesets was positively correlated with 33 out of the 38 probesets suggesting a high degree of co-regulation of SYK, SYK dependent STAT3 targets and anti-apoptotic genes in leukemia samples. These findings confirm and extend our earlier observations regarding the role of SYK as a regulator of the anti-apoptotic STAT3 signaling pathway (Uckun et al., 2010a). We next examined archived GEP data on initial diagnostic bone marrow samples from 48 BPL patients, who experienced an early relapse < 36 months after diagnosis vs. 28 who experienced a late relapse ≥ 36 months after diagnosis, to determine if their expression levels of the SYK-STAT3 pathway genes differed (2-sample T-test, unequal variance correction). Probesets for KLF4 (2.26 fold difference, T-test P-value = 0.0177), BIRC5 (2 probesets with 1.65 and 1.64 fold differences with P values of 0.0006 and 0.0032 respectively), HSPA5 (1.60 fold difference, P-value = 0.0169), DAD1 (1.58 fold difference, P-value = 0.0130) and SYK (1.49 fold difference, P-value = 0.0196) exhibited significantly increased expression levels in leukemia cells from patients who experienced an early relapse vs. leukemia cells from patients who experienced a late relapse (Fig. 1B). The identification of these SYK-regulated genes as biomarkers for subpopulations of patients who are at high risk for treatment failure and early relapse is in accord with our earlier studies that established SYK as a molecular target for treatment of relapsed BPL (Uckun et al., 2010a, Uckun et al., 2010b, Uckun et al., 2013, Uckun and Qazi, 2010).

### 3.2 Liposomal Nanoparticles of the SYK P-Site Inhibitor C61 (C61-LNP) Plus Low Dose Radiation Kills Leukemia-Initiating Cells (LIC) in BPL Xenograft Samples

**Fig. 2:**
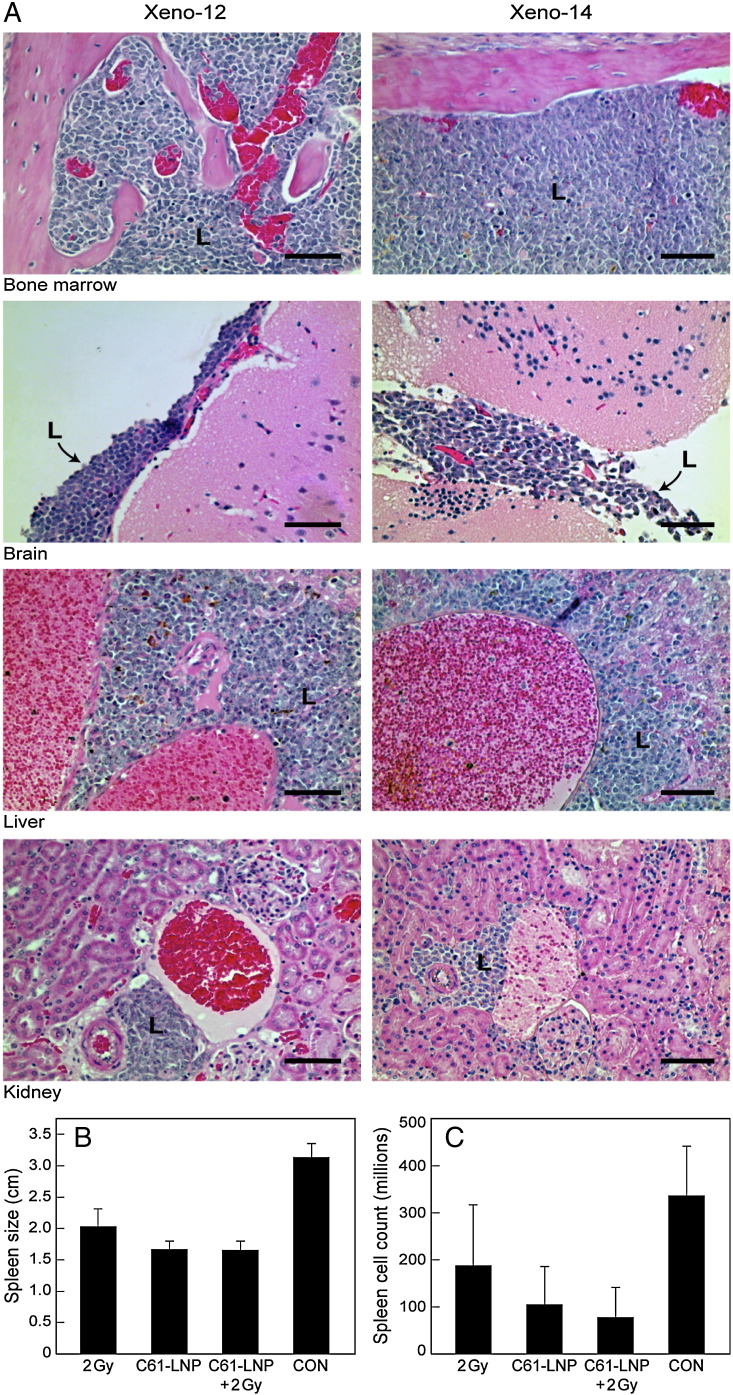
Anti-leukemic effects of C61-LNP + radiation against leukemia-initiating in vivo clonogenic BPL xenograft cells. Xenograft cells isolated from spleens of leukemic NOD/SCID mice were treated with C61-LNP (C61 concentration: 30 μg/mL), 2 Gy γ-rays, C61-LNP + 2 Gy γ-rays or left untreated for 24 h at 37 °C and then reinjected into NOD/SCID mice (150,000 cells/mouse). [A] Histopathological evidence of disseminated leukemia in NOD/SCID mice challenged with human BPL xenograft cells derived from relapsed BPL patients. NOD/SCID mice challenged with untreated BPL xenograft cells rapidly developed disseminated leukemia. At the time of death, necropsies were performed and revealed massive splenomegaly with an average spleen size of 3.35 ± 0.11 cm and nucleated spleen cell count of 447 (± 236) × 10^6^. Depicted are the histopathological results for select organs in control mice from both xenograft cases (Xeno-12 and Xeno-14). Bone marrow involvement was manifested as replacement of normal tissue elements by diffuse sheets of densely packed leukemic cells. Infiltrated kidneys showed cortical, interstitial and perivascular accumulations of leukemic cells. Livers showed leukemic infiltrates in the portal spaces and sinusoids. The leptomeninges of the brain contained rafts of leukemic cells. Images were taken with an EVOS XL Core Light Microscope (AMG, Bothell, WA) using a 40 × objective. L: leukemic infiltrate. Scale bar: 100 μm. [B] Depicted are the spleen sizes of NOD/SCID mice challenged with xenograft cells exposed to various treatments. NOD/SCID mice challenged with untreated xenograft ALL cells rapidly developed overt leukemia with massive splenomegaly. The average spleen size was 3.14 ± 0.22 cm (“massive enlargement”) for the control group (N = 12), 2.04 ± 0.28 cm for the radiation alone group (N = 11; Linear Contrast P-value = 0.0003), 1.68 ± 0.12 cm for the C61-LNP alone group (N = 12; P-value < 0.0001), and 1.66 ± 0.14 cm for the C61-LNP + radiation group (N = 14; P-value < 0.0001). [C] Depicted are the spleen nucleated cell counts of NOD/SCID mice challenged with xenograft cells exposed to various treatments. The average spleen nucleated cell count (in millions) as a measure of the leukemic burden was 337 ± 103 (log_10_ = 2.3 ± 0.14) for the control group (N = 12), 188 ± 129 (log_10_ = 1.46 ± 0.24; Linear Contrast P-value = 0.0038) for the 2 Gy radiation alone group (N = 11), 106 ± 81 (log_10_ = 1.36 ± 0.18; P-value = 0.0011) for the C61-LNP alone group (N = 12), and 78 ± 63 (log_10_ = 1.1 ± 0.17; P-value < 0.0001) for the C61-LNP + radiation group.

We first compared the in vitro anti-leukemic activity of low dose (2 Gy) radiation alone, C61-LNP alone and C61-LNP in combination with low dose radiation against the in vivo clonogenic LIC fraction in xenograft specimens from 2 relapsed BPL patients using a NOD/SCID mouse xenograft model of BPL (Fig. 2). Mice challenged with xenograft cells that were treated with C61-LNP + radiation had the smallest size (albeit not significantly different from the C61-LNP alone or radiation alone groups: P-value = 0.97, vs. C61-LNP; P-value = 0.18, vs. radiation alone) and the lowest leukemia burden (albeit not significantly different from the C61 alone or radiation alone groups: P-value = 0.32, vs. C61-LNP; P-value = 0.19, vs. radiation alone) when compared to all other groups of mice. These findings demonstrate that C61-LNP alone as well as in combination with low dose radiation damages the in vivo clonogenic LIC fraction in xenograft cell populations derived from patients with aggressive BPL and abrogates their ability to engraft and cause overt leukemia in NOD/SCID mice. The potent single agent anti-leukemic potency of C61-LNP against LIC did not allow a more accurate and detailed assessment of potential additive or synergistic effects between C61-LNP and radiation in vitro.

### 3.3 C61-LNP Formulation Augments the Anti-Leukemic Potency of Low Dose TBI and Improves the Survival Outcome in NOD/SCID Mouse Models of Relapsed BPL

**Fig. 3:**
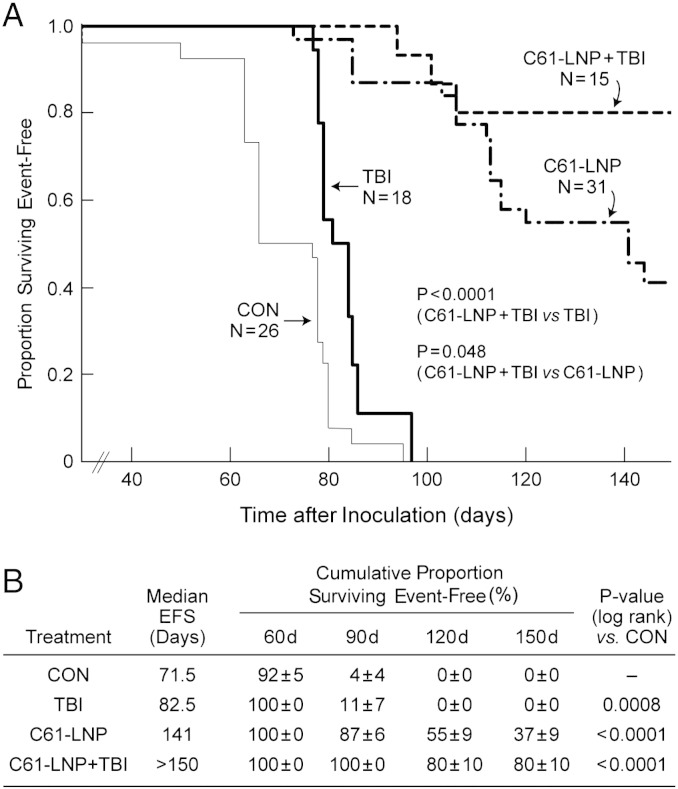
In vivo anti-leukemic efficacy of C61-LNP + TBI in a NOD/SCID mouse xenograft model of relapsed BPL. We compared the efficacy of C61-LNP and C61-LNP + TBI to TBI alone in 2 BPL xenograft models (viz.: Xeno-12 and Xeno-14) derived from primary leukemia cells of 2 pediatric patients with relapsed BPL. The xenograft cells from Xeno-12 were CD19^+^ (99.6%), CD10^+^CD34^+^ (98.9%), CD19^+^CD45^+^ (99.6%) and CD19^+^CD34^+^ (98.9%), and SYK^+^ (76% by intracytoplasmic flow cytometry) (Uckun et al., 2013). The xenograft cells from Xeno-14 were CD19^+^ (98.8%), CD10^+^CD34^+^ (68.2%), CD19^+^CD45^+^ (92.0%), CD19^+^CD34^+^ (70.4%), and SYK^+^ (97.1%) by intracytoplasmic flow cytometry (Uckun et al., 2013). C61-LNP was administered either alone or in combination with TBI according to two schedules: (i) 3-day schedule = Days 1–3: C61-LNP, 80 mg/kg/day i.v. 2 Gy single dose TBI was administered on Day 2 in the C61-LNP + TBI group; (ii) 5-day schedule = Days 1–5: C61-LNP, 80 mg/kg/day i.v. 2 Gy single dose TBI was administered on Day 5. Controls included untreated mice and mice treated with D5W. Depicted are the event-free survival curves (A) and life table statistics for NOD/SCID mice subjected to different treatment regimens. Shown are the cumulative data on all mice treated according to a 3-day or 5-day schedule: C61-LNP: Of 31 mice, 19 were treated according to the 3 day schedule while 12 were treated according to the 5 day schedule. C61-LNP + TBI: Of the 15 mice, 3 were treated according to the 3 day-schedule and 12 were treated according to the 5 day-schedule. Notably, the C61-LNP + TBI combination was more effective than both TBI alone (Log Rank P-value: < 0.0001) and C61-LNP alone (Log Rank P-value: 0.048). This unique combination regimen resulted in a median EFS time of > 150 days and a remarkable 150-day leukemia-free survival of 80 ± 10%. All fatal events (spontaneous death or sacrifice) were related to overt leukemia. C61-LNP as well as C61-LNP + TBI treatments were well tolerated without treatment related morbidity or mortality. No treatment-related toxic lesions were found by histopathological examination in any of the tissues of the mice that developed fatal leukemia in the C61-LNP or C61-LNP + TBI groups.

We next set out to determine if C61-LNP can augment the anti-leukemic potency of TBI against human BPL cells in vivo using our NOD/SCID mouse xenograft model of relapsed BPL. All 26 untreated (N = 16) or D5W-treated (N = 10) control NOD/SCID mice challenged with an intravenous inoculum of 1 or 2 × 10^6^ radiation-resistant ALL xenograft cells derived from relapsed BPL patients either died or were killed in moribund condition due to their advanced leukemia within 95 days with a median event-free survival (EFS) time of only 71.5 days (Fig. 3). Likewise, all 18 mice treated with 2 Gy single dose TBI alone developed fatal leukemia within 97 days with a median EFS time of 82.5 days. In contrast, the median EFS time was prolonged to 141 days with a 37 ± 9.6% 150 day EFS rate for the 31 test mice that were treated with 3 or 5 daily i.v. injections of C61-LNP (daily dose: 80 mg/kg). Thus, the C61-LNP treatment regimen resulted in a significant improvement of the EFS outcome in NOD/SCID mice challenged with an invariably fatal dose of patient-derived BPL xenograft cells (P-value: < 0.0001 for C61-LNP vs. control and C61-LNP vs. TBI comparisons) (Fig. 3). Notably, the C61-LNP + TBI combination (N = 15) was more effective in vivo than TBI alone (Log Rank P-value: < 0.0001) or C61-LNP alone (Log Rank P-value: 0.048). This unique combination regimen resulted in a median EFS time of > 150 days and a remarkable 150-day leukemia-free survival of 80 ± 10%. C61-LNP as well as C61-LNP + TBI treatments were well tolerated without treatment related morbidity or mortality.

### 3.4 C61-LNP Plus Low Dose TBI Improves the Survival Outcome in a CD22ΔE12 × BCR–ABL Double Transgenic (Tg) Model of Advanced Murine BPL

**Fig. 4:**
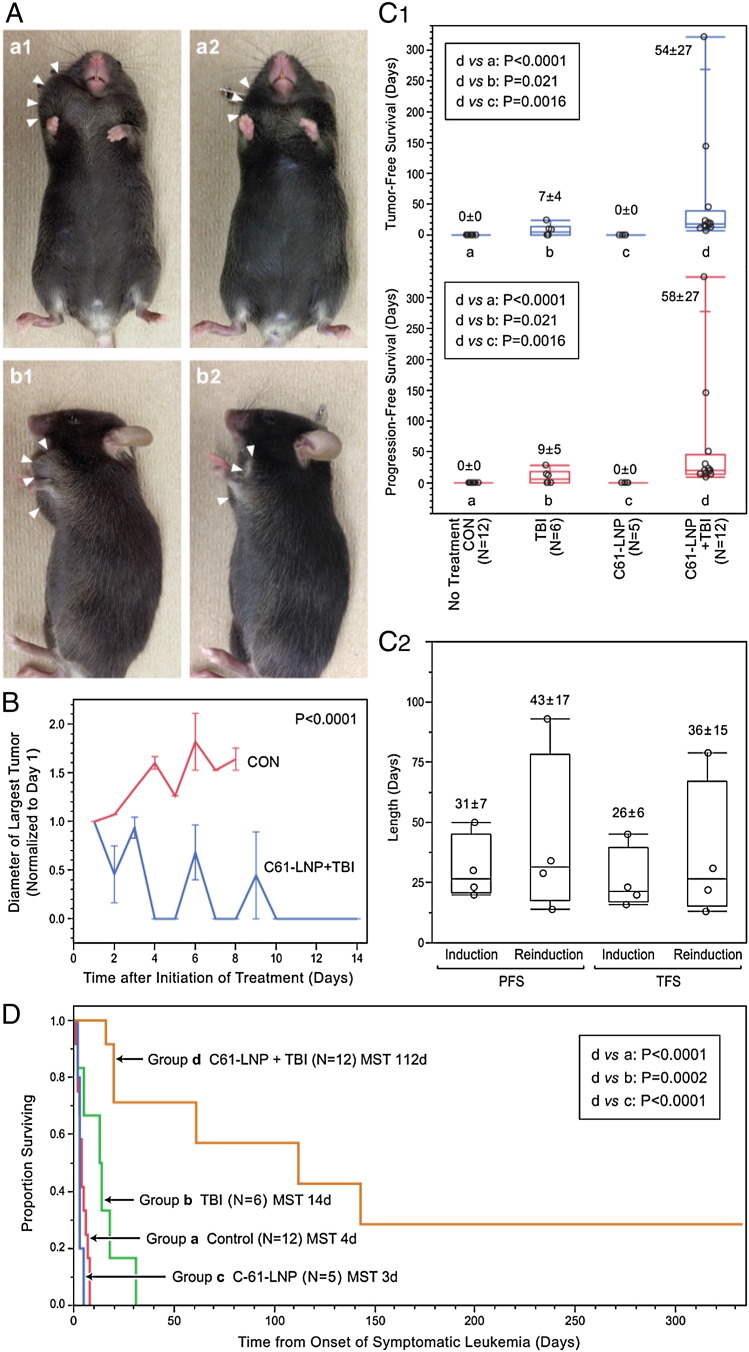
Anti-leukemic activity of C61-LNP plus low dose TBI against murine B-precursor leukemia in CD22ΔE12 × BCR–ABL double-Tg mice. [A] Depicted are the Day 1 vs. Day 7 images of representative CD22ΔE12 × BCR–ABL double-Tg mice assigned to different treatment protocols. The initial observable target tumor size was comparable among groups, as measured by the longest diameter: 1.0 ± 0.1 cm for CON (N = 8), 1.0 ± 0.1 cm for 4 Gy TBI (N = 5), 1.4 ± 0.3 cm for 80 mg/kg C61-LNP (N = 5), and 1.0 ± 0.1 (N = 11) cm for 80 mg/kg C61-LNP + 4 Gy TBI (N = 11) (One-way ANOVA across treatments, P = 0.26) No tumor measurements were available in 4 control mice, one mouse treated with TBI alone and one mouse treated with C61-LNP + TBI. These mice had symptomatic systemic leukemia. [B] Tumor sizes were based on the longest diameter of the largest visible target tumor and normalized to the pre-treatment tumor size on Day 1. Depicted are the values for normalized tumor size during the first 14 days after initiation of treatment. The repeated measures analysis of co-variance model explained significant proportion of the variance in the growth profiles with a highly significant difference between CON and C61-LNP + TBI groups (repeated measures ANOVA, 86% of the variance explained, F_1,14.1_ = 51.7, P < 0.0001). The fluctuation in the average tumor volume of the test group seen on Days 6 and 9 is caused by the data obtained in one mouse. This particular mouse had a slow remission induction after C61-LNP + TBI (tumor was reduced in size but still detectable on Days 6 and 9). This mouse is still alive in remission at 334 days (tumor free survival 322 days +). [C1] Tumor-free survival (TFS in days) and progression-free survival (PFS in days) distributions are depicted for the induction treatment using quantile dot plots (box represents 25th, median and 75th percentiles, and the whiskers represent the minimum, 10th, 90th and maximum values). Pair-wise differences in median survival times were calculated using non-parametric Wilcoxon tests. [C2] Tumor-free survival (TFS in days) and progression-free survival (PFS in days) distributions are depicted for matched pair the induction vs. reinduction treatments of relapsing mice using quantile dot plots (box represents 25th, median and 75th percentiles, and the whiskers represent the minimum, 10th, 90th and maximum values). Pair-wise differences in median survival times were calculated using non-parametric Wilcoxon tests. [D] The survival outcome as measured by the number of days the mice remained alive after the onset of symptomatic leukemia/1st day is depicted by interval curves generated using the Kaplan–Meier product limit method and the P-values were calculated using log-rank tests. C61-LNP + TBI treatment exhibited significant improvements in all treatment response parameters measured compared to all other treatment groups. Shown are the cumulative data on all mice regardless of schedule of the C61-LNP. In the C61-LNP alone group, all mice experienced rapid progression of leukemia while receiving treatments. Therefore, they could only receive 2 (one mouse), 3 (2 mice) or 4 doses (2 mice) of the intended 9 doses of C61-LNP until they died or were terminated according to humane treatment criteria. In the C61-LNP + TBI group, 6 mice received a single dose of C61-LNP (80 mg/kg/dose) during frontline therapy and 6 mice received multiple doses of C61-LNP (80 mg/kg/dose) of which 3 were assigned to 3 doses and 3 were assigned to 9 doses. TBI (4 Gy) was administered as a single dose 1 h after injection of C61-LNP on Day 3 in the multiple dose regimen or on day 1 in the single dose regimen.

CD22ΔE12 × BCR–ABL double-Tg mice spontaneously develop highly radiation-resistant fatal BPL with lymphomatous features at a median of 78 days (Uckun et al., 2015). Mice were randomly assigned to one of 4 treatment protocols when they developed symptomatic leukemia with measurable tumor masses. As shown in Fig. 4, the combination of C61-LNP (80 mg/kg based on C61 content) with 4 Gy TBI yielded progression-free survival (PFS) and tumor-free survival (TFS) outcomes significantly superior to those of untreated control mice (CON) or mice treated with TBI alone or C61-LNP alone. We observed a greater than 1.5-fold increase of tumor size in CON mice after 4 days, whereas the tumors of the mice in the C61-LNP + TBI group rapidly regressed within 4 days and remained undetectable or very small for > 14 days after each course of treatment (Fig. 4A & B). The mean ± SE values for PFS were 58 ± 27 days for C61-LNP + TBI, but only 0 ± 0 days for CON (non-parametric Wilcoxon test, P < 0.0001), 0 ± 0 days for C61-LNP alone (P = 0.0016), and 9 ± 5 days for TBI (P = 0.021) (Fig. 4C). Six of 6 mice treated with a single dose of C61-LNP (80 mg/kg based on C61 content) + single dose TBI (4 Gy) and 6 of 6 mice treated with 3 doses of C61-LNP (80 mg/kg) + single dose TBI (4 Gy) rapidly achieved a remission (time to remission: 4.1 ± 0.9 (SEM) days). By comparison, only 3 of 6 mice treated with TBI alone (Fisher's Exact test, 2-tailed comparing TBI alone with TBI + C61-LNP combination, P = 0.02) and none of the 8 mice treated with C61-LNP alone achieved remission (P < 0.0001). The duration of remission as measured by the TFS times were 54 ± 27 days for C61-LNP + TBI, but only 7 ± 4 days for TBI alone (non-parametric Wilcoxon test, P = 0.021) and 0.0 ± 0.0 days for CON (P < 0.0001) or C61-LNP alone (P = 0.0016) (Fig. 4C). 4 mice on the combination regimen relapsed and rapidly achieved a second remission after a single dose of C61-LNP + TBI. Thus, their initial treatment failure was caused by suboptimal dosing in the context of a large tumor burden rather than intrinsic resistance to the combination therapy. The overall survival (OS) outcome as measured by the number of days the mice remained alive after the onset of symptomatic leukemia/1st day of treatment showed a markedly improved outcome after C61-LNP + TBI vs. TBI alone or C61-LNP alone. The median OS times were 112 days for C61-LNP + TBI but only 4 days for CON (Log-rank test, P < 0.0001), 3 days for C61-LNP alone (P < 0.0001), and 14 days for TBI alone (P = 0.0002) (Fig. 4D).

## 4 Discussion

TBI-based conditioning regimens have not prevented leukemic relapses in high-risk BPL patients post-HSCT (Gaynon et al., 2006, Balduzzi et al., 2014). In agreement with the radiation resistance of BPL cells, the risk of post-HSCT relapse is particularly high in patients with a high residual leukemia burden prior to TBI (Pulsipher et al., 2009, Bar et al., 2014). The development of new drugs that can help overcome the radiation resistance of BPL cells would be an important step forward in efforts aimed at improving the post-HSCT outcomes. The identification of SYK as a regulator of the anti-apoptotic STAT-3 response to oxidative stress (Uckun et al., 2010a) prompted the hypothesis that tyrosine kinase inhibitors targeting SYK may overcome the resistance to oxidative stress-induced apoptosis and thereby provide the foundation for more effective TBI-based pretransplant conditioning regimens for poor prognosis BPL patients undergoing HSCT.

Our study provides the preclinical proof-of-concept that the C61-LNP plus low dose TBI would be a safe and effective treatment modality for the treatment of relapsed BPL. The unique combination of C61-LNP with 2 Gy TBI in NOD/SCID mice or 4 Gy TBI in CD22ΔE12 × BCR–ABL double transgenic mice was very well tolerated without any treatment related morbidity or toxic deaths. The demonstrated ability of C61-LNP to markedly augment the anti-leukemic potency of low dose TBI, induce remissions and improve the EFS outcome in two separate models of radiation-resistant BPL without serious added toxicity provides the preclinical proof of concept for C61-LNP plus reduced intensity TBI as a new conditioning regimen that has the potential to significantly improve the EFS outcome and long-term health status of high-risk remission BPL patients undergoing HSCT. We hypothesize that the incorporation of C61-LNP into the pre-transplant TBI regimens of patients with relapsed or very high-risk BPL will improve their survival outcome after HSCT.

## Disclosure of Potential Conflicts of Interest

No potential conflicts of interest were disclosed.

## Grant Support

F.M.U. was supported in part by 10.13039/100005561DHHS grants P30CA014089, U01-CA-151837, R01CA-154471 and R21-CA-164098 from the 10.13039/501100002387National Cancer Institute. J.C. was supported by the NIH Director's New Innovator Award 1DP2OD007246. The content is solely the responsibility of the authors and does not necessarily represent the official views of the National Cancer Institute or the National Institutes of Health.

## Author Contributions

All authors have made significant and substantive contributions to the study. All authors reviewed and revised the paper. F.M.U. was the NIH-funded Principal Investigator who designed, directed and supervised this study and wrote the initial draft of the manuscript. S.Q. performed the statistical analyses and PK parameter determinations. J.C., D.E.M. and F.M.U. performed experiments, acquired data, analyzed data, interpreted data and revised the initial draft manuscript.
