# Supplementary material for: Liposomal Nanoparticles of a Spleen Tyrosine Kinase P-Site Inhibitor Amplify the Potency of Low Dose Total Body Irradiation Against Aggressive B-Precursor Leukemia and Yield Superior Survival Outcomes in Mice
Source: eBioMedicine. 2015 Apr 11;2(6):554–62. doi: 10.1016/j.ebiom.2015.04.005 (PMC4535154; doi:10.1016/j.ebiom.2015.04.005)
Supplement: Supplementary file 1 — Supplementary material. [file mmc1.doc]

**Supplement**

**Physicochemical Characterization of Nanoparticles.**  We measured the size of the C61-LNP by using the dynamic light scattering (DLS) technique. The C61 content of the LNP was measured using analytical HPLC. Transmission electron microscopy (TEM) on the C61-LNP was performed using a JEOL JEM-2100 LaB6 (Peabody, MA) electron microscope.


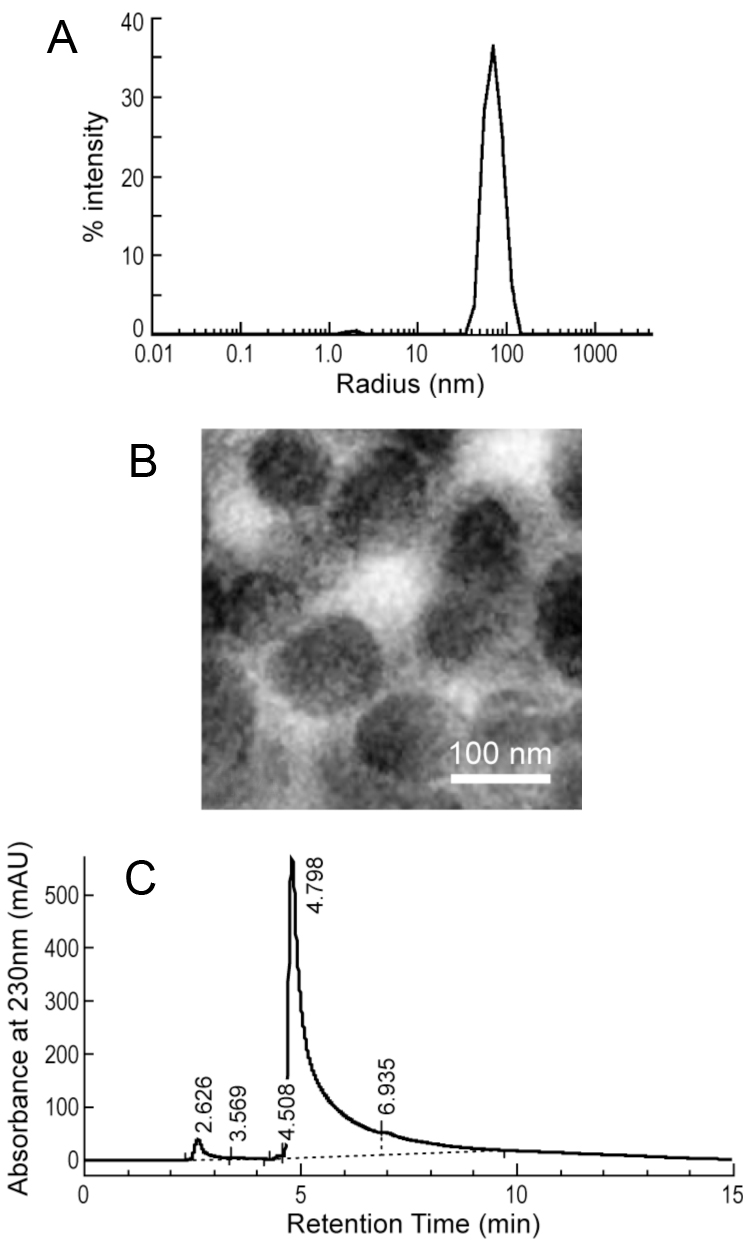


**Figure S1. Characteristics of the C61-LNP 25A.** [A] Particle size (radius) measurement of 25A nanoparticles using dynamic light scattering (DLS). The average (mean±SEM) diameter of the 25 nanoparticles was 136.3±1.2 nm (N=15). [B] Transmission electron microscopy (TEM) images of 25A nanoparticles showing an ellipsoid-spherical shape. [C] Detection of C61 in 25A using HPLC. The average (mean±SEM) C61 concentration was 8.7±0.1 mg/mL (N=11). Modified from the supplemental information associated with the publication: Uckun et al., [Nanoscale liposomal formulation of a SYK P-site inhibitor against B-precursor leukemia.](http://www.ncbi.nlm.nih.gov/pubmed/23568490) Blood. 2013; 121: 4348-54.
